# Antioxidant Peptides from Miiuy Croaker Swim Bladders: Ameliorating Effect and Mechanism in NAFLD Cell Model through Regulation of Hypolipidemic and Antioxidant Capacity

**DOI:** 10.3390/md23020063

**Published:** 2025-02-01

**Authors:** Yu-Mei Wang, Ming-Xue Ge, Su-Zhen Ran, Xin Pan, Chang-Feng Chi, Bin Wang

**Affiliations:** 1 Zhejiang Provincial Engineering Technology Research Center of Marine Biomedical Products, School of Food and Pharmacy, Zhejiang Ocean University, Zhoushan 316022, China; wangym731@126.com (Y.-M.W.);; 2 School of Foundation Studies, Zhejiang Pharmaceutical University, Ningbo 316022, China; 3 National and Provincial Joint Laboratory of Exploration, Utilization of Marine Aquatic Genetic Resources, National Engineering Research Center of Marine Facilities Aquaculture, School of Marine Science and Technology, Zhejiang Ocean University, Zhoushan 316022, China

**Keywords:** miiuy croaker (*Miichthys miiuy*) swim bladder, FSGLR, GIEWA, nonalcoholic fatty liver disease (NAFLD), hypolipidemic effect, antioxidant activity, molecular docking

## Abstract

In this work, the hypolipidemic and antioxidative capacity of FSGLR (S7) and GIEWA (S10) from miiuy croaker swim bladders was explored systematically in an oleic acid (OA)-induced nonalcoholic fatty liver disease (NAFLD) model of HepG2 cells. Moreover, the hypolipidemic activity of S7 and S10 and their antioxidative abilities were preliminarily investigated in combination with molecular docking technology. The results indicated that S7 and S10 could decrease the amount of lipid accumulation and the content of triglycerides (TG) and total cholesterol (TC) in the OA-induced NAFLD cell model in a dose-dependent manner. In addition, S7 and S10 exhibited better bile salt binding, pancreatic lipase (PL) inhibition, and cholesterol esterase (CE) inhibition capacities. The hypolipidemic mechanisms of S7 and S10 were connected with the downregulation of the mRNA expression levels of adipogenic factors, including sterol-regulatory element-binding protein-1c (SREBP-1c), acetyl-CoA carboxylase (ACC), sterol-regulatory element-binding protein (SREBP)-2, hydroxymethylglutaryl-CoA reductase (HMGR), and fatty acid synthase (FAS) (*p* < 0.01), and the upregulation of the mRNA expression of β-oxidation-related factors, including carnitine palmitoyltransferase 1 (CPT-1), acyl-CoA oxidase 1 (ACOX-1), and peroxisome proliferator-activated receptor α (PPARα). Moreover, FSGLR (S7) and GIEWA (S10) could significantly protect HepG2 cells against OA-induced oxidative damage, and their antioxidant mechanisms were related to the increased activity of intracellular antioxidant proteases (superoxide dismutase, SOD; glutathione peroxidase, GSH-PX; catalase, CAT) to remove excess reactive oxygen species (ROS) and decrease the production of malondialdehyde (MDA). The presented findings indicate that the hypolipidemic and antioxidant functions and mechanisms of S7 and S10 could make them potential hypolipidemic and antioxidant candidates for the treatment of NAFLD.

## 1. Introduction

Nonalcoholic fatty liver disease (NAFLD) is a severe and widespread liver disease that is characterized by increased intrahepatic triglyceride (TG) and total cholesterol (TC) accumulation, steatosis, liver inflammation, hepatocellular damage, and progressive fibrosis [1,2]. Notably, NAFLD is strongly associated with metabolic disturbances, such as hyperglycemia, central obesity, insulin resistance, lipid metabolism disorder, adult-onset diabetes, high blood pressure, and persistent abnormal liver function [3]. At present, NAFLD affects approximately 25% of the global adult population, exerting a sustained negative impact on public health and creating a wide range of social and economic burdens [1,3].

Normally, lipid metabolism exists in a dynamic equilibrium between lipid synthesis and digestion in the liver, but disruptions to this metabolic balance will cause lipid accumulation in liver cells and further lead to oxidative stress and hepatic steatosis [4,5]. Lipid accumulation (or lipotoxicity), oxidative stress, and inflammation exert critical impacts during the subsequent development of NAFLD [6,7]. Therefore, reducing excess liver fat is considered an effective strategy to control the onset of NAFLD, and the elimination or amelioration of oxidative injury caused by reactive oxygen species (ROS) also plays an essential role in controlling NAFLD [8]. Behavioral and lifestyle changes, including weight loss, diet control, and physical exercise, are important ways to manage NAFLD, especially to reduce lipid accumulation. However, achieving and maintaining this level of weight loss is extremely difficult for most NAFLD patients through lifestyle adjustments alone [9]. Therefore, synthetic drugs, such as fenofibrate and pioglitazone, are considered to treat NAFLD, but these drugs have some side effects that should not be ignored [5,10]. Therefore, new classes of drugs, such as farnesoid X receptor (FXR) agonists, glucagon-like peptide 1 (GLP-1) agonists, and thyroid hormone receptor-β (THR-β) agonists, are emerging for the treatment of NAFLD, nonalcoholic steatohepatitis (NASH), and fibrosis [9].

Bioactive peptides (BPs) have been produced from diverse plant and animal proteins, such as mammals [11], poultry [12], beans [13], mollusks [14,15], fish [16,17], Antarctic krill [18], algae [19], etc. Beyond their important nutritional benefits, BPs show remarkable bioactivity and pharmacological functions, with numerous potential applications for human health [20,21,22]. Notably, some BPs exhibit hypolipidemic, antioxidant, and anti-inflammatory activity and show obvious advantages in the treatment of NAFLD [23,24,25]. For example, GINY and DQW from α-lactalbumin could alleviate NAFLD by activating the peroxisome proliferator-activated receptor α (PPARα) pathway to decrease lipid deposition and oxidative stress [26,27]. VLATSGPG (VLA) from salmon skins could decrease the lipid content and inflammation in free fatty acid (FFA)-damaged HepG2 cells by regulating the PERK/eIF2α and PERK/IκBα pathways [28]. A tetrapeptide (VHVV) from soy exhibited a strong preventive effect on liver dysfunction caused by hyperglycemia [29]. The β-conglycinin (βCG) peptide derived from soy could decrease the quantity and content of white adipose tissue in the abdomens of Otsuka Long-Evans Tokushima fatty (OLETF) rats by controlling lipogenic and lipolytic enzymes’ activity [30]. A tripeptide of RPR derived from protamine showed noteworthy anti-obesity and hypocholesterolemic activity and played a vital role in the anti-obesity action in high-fat diet (HFD)-induced C57BL/6J mice because it could lower the serum cholesterol level, reduce the white adipose tissue weight, and increase the excretion of TC and bile acids in the stool [31]. The hypolipidemic peptides of FLF, IYF, and QIF from tea proteins presented prominent cholesterol esterase-inhibitory activity, pancreatic lipase-inhibitory abilities, and sodium taurocholate-binding capacities [32]. Consequently, BPs can serve as functional foods, nutritional supplements, and pharmaceutical products to treat NAFLD.

The swim bladder is a by-product of fish processing and is often used as a low-value feed material. However, swim bladders can also serve as high-quality raw materials to generate functional components, such as collagens/gelatins [33,34,35], protein hydrolysates, BPs [36,37,38,39], and polysaccharides/glycosaminoglycans [40,41,42]. Moreover, using fish by-products to develop bioactive ingredients or products is a good strategy to reduce economic losses, protect the ecological environment, and provide consumers with high-quality products [43]. Therefore, ten antioxidant BPs, namely FYKWP (S1), FTGMD (S2), GFEPY (S3), YLPYA (S4), FPPYERRQ (S5), GFYAA (S6), FSGLR (S7), FPYLRH (S8), VPDDD (S9), and GIEWA (S10), were separated from the swim bladder hydrolysate of the miiuy croaker in our previous work [38,44], and it is worth noting that FSGLR (S7) and GIEWA (S10) showed significant inhibitory abilities regarding lipid accumulation, as well as antioxidant functions, in oleic acid (OA)-induced HepG2 cells. Therefore, the present study’s objective was to systematically explore the hypolipidemic and antioxidant capacities of FSGLR (S7) and GIEWA (S10) in an OA-induced NAFLD model of HepG2 cells.

## 2. Results

### 2.1. Hypolipidemic Capacity of FSGLR (S7) and GIEWA (S10) in OA-Induced NAFLD Cell Model

#### 2.1.1. Effects of BPs (S1-S10) on Viability of HepG2 Cells

**Figure 1:**
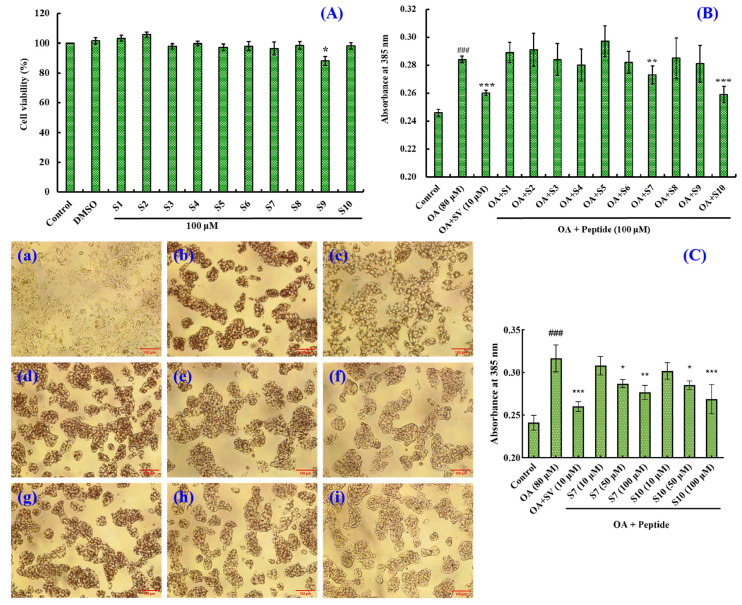
The viability of HepG2 cells incubated with antioxidant peptides (S1–S10) at 100 μM. (**A**) * *p* < 0.05 vs. control group. Effects of antioxidant peptides (S1–S10) at 100 μM on intracellular lipid accumulation in OA-induced HepG2 cells. (**B**) ^###^ *p* < 0.001 vs. control group; *** *p* < 0.001 and ** *p* < 0.01 vs. model group. Oil Red O staining: Effects of FSGLR (S7) and GIEWA (S10) on OA-induced HepG2 cell morphology as well as intracellular lipid accumulation. (**C**) a: Control; b: Model (OA); c: Simvastatin; d: S7 (10 μM); e: S7 (50 μM); f: S7 (100 μM); g: S10 (10 μM); h: S10 (50 μM); i: S10 (100 μM). ^###^ *p* < 0.001 vs. control group; *** *p* < 0.001, ** *p* < 0.01, and * *p* < 0.05 vs. model group.

Figure 1A shows that the viability of HepG2 cells incubated with antioxidant peptides (S1–S10) at 100 μM ranged from 88.2% ± 3.08% to 105.68% ± 1.71%. However, their viability when incubated with VPDDD (S9) was remarkably lower than that of the control (*p* < 0.05), which indicated that VPDDD (S9) had cytotoxic activity and could inhibit the HepG2 cells’ proliferation at 100 μM. Therefore, these ten antioxidant peptides (S1-S10), with the exception of VPDDD (S9), could be applied in the development of functional products.

#### 2.1.2. Effects of BPs (S1–S10) on Lipid Accumulation in OA-Induced NAFLD Cell Model

Figure 1B shows that the lipid quantity in OA-induced HepG2 cells was considerably higher than that in the control (*p* < 0.001), indicating that the OA-induced NAFLD cell model was successfully established. At 100 μM, FSGLR (S7) and GIEWA (S10) could dramatically reduce the lipid quantity in OA-induced HepG2 cells (*p* < 0.01 or 0.001). Moreover, GIEWA (S10) showed a similar effect to simvastatin (SV) in decreasing lipid accumulation. This finding is confirmed by the images in Figure 1C, where darker and more numerous lipid droplets indicate more severe lipid accumulation, and Oil Red O staining showed higher lipid content and more lipid droplets in the cells of the model group compared to the control group. However, the number of lipid droplets was significantly increased after the administration of moderate or high concentrations of S7 and S10. In addition, the absorbance of FSGLR (S7) and GIEWA (S10) at 100 μM was 0.277 ± 0.007 and 0.269 ± 0.014, respectively, and the absorbance of the SV group at 10 μM was 0.260 ± 0.005. Obviously, FSGLR (S7) and GIEWA (S10) at 100 μM and SV at 10 μM could dramatically reduce the majority of the cellular lipids (*p* < 0.01 or 0.001), and S10 and SV were more effective. The present results demonstrate that FSGLR (S7) and GIEWA (S10) are good hypolipidemic candidates, and their mechanisms will be discussed in the following sections.

#### 2.1.3. Effects of FSGLR (S7) and GIEWA (S10) on Hypolipidemic Activity

**Figure 2:**
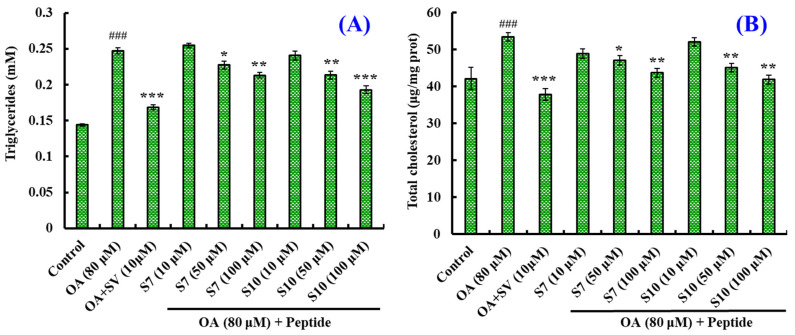
Effects of FSGLR (S7) and GIEWA (S10) on TG (**A**) and TC (**B**) content in OA-induced NAFLD cell model. ^###^ *p* < 0.001 vs. control group; *** *p* < 0.001, ** *p* < 0.01, and * *p* < 0.05 vs. model group. The cells were collected and lysed, and then the TG and TC content was determined.

Figure 2 illustrates that FSGLR (S7) and GIEWA (S10) could dose-dependently decrease the TG and TC content of OA-induced HepG2 cells. Compared with the model group, the levels of TG and TC in the FSGLR (S7) and GIEWA (S10) groups were not significantly different at a low concentration (10 μM) (*p* > 0.05), but they significantly decreased at a medium concentration (50 μM) (*p* < 0.05 or < 0.01). At 100 μM, the TG content in the FSGLR (S7) and GIEWA (S10) groups decreased to 0.213 ± 0.004 and 0.193 ± 0.005 mM, and the TC content in the FSGLR (S7) and GIEWA (S10) groups decreased to 43.69 ± 1.24 and 41.90 ± 1.23 μg/mg prot. These data were observably smaller than those observed in model cells (*p* < 0.01 or 0.001). Furthermore, the TG and TC content of the GIEWA (S10) group was inferior to that of the FSGLR (S7) group. The presented data suggest that FSGLR (S7) and GIEWA (S10) could decrease the content of TC and TG in OA-induced HepG2 cells.

**Figure 3:**
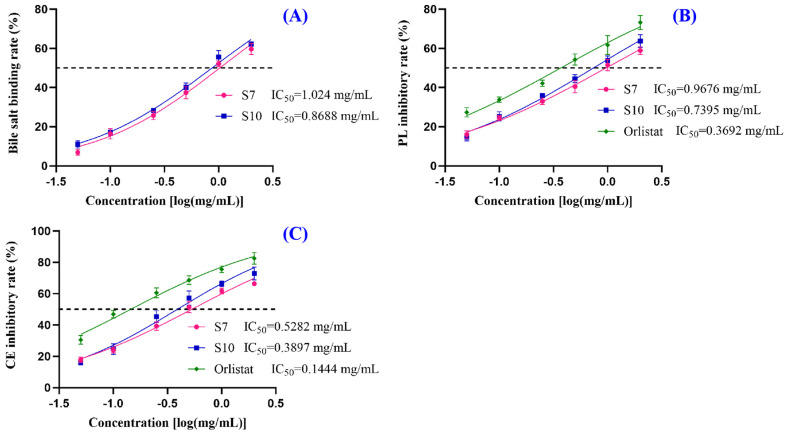
Effects of FSGLR (S7) and GIEWA (S10) on in vitro hypolipidemic activity, including bile salt binding rate (**A**), pancreatic lipase (PL) inhibitory activity (**B**), and cholesterol esterase (CE) inhibitory activity (**C**).

To further investigate the mechanism by which FSGLR (S7) and GIEWA (S10) reduced the TG and TC levels, the bile salt binding rate, pancreatic lipase (PL) inhibitory activity, and cholesterol esterase (CE) inhibitory activity of FSGLR (S7) and GIEWA (S10) were assayed. The bile salt binding rates of the different peptides are shown in Figure 3A. With an increase in the FSGLR (S7) and GIEWA (S10) concentrations, the bile salt binding rate increased in a dose-dependent manner, and the IC_50_ value of GIEWA (S10) reached 0.8688 mg/mL, which was better than that of FSGLR (S7) (IC_50_: 1.024 mg/mL). Orlistat binds to and inactivates lipase in the gastrointestinal tract, thereby inhibiting lipolysis and reducing fat absorption. Figure 3B shows that GIEWA (S10) and FSGLR (S7) could dose-dependently inhibit the activity of pancreatic lipase (PL), with IC_50_ values of 0.7395 and 0.9676 mg/mL, respectively. However, the inhibition rates of GIEWA (S10) and FSGLR (S7) were lower than that of Orlistat (IC_50_: 0.3692 mg/mL). Figure 3C illustrates that the IC_50_ values of GIEWA (S10) and FSGLR (S7) for cholesterol esterase (CE) were 0.3897 and 0.5282 mg/mL, respectively. Moreover, the inhibitory activity of GIEWA (S10) was similar to that of Orlistat (IC_50_: 0.1444 mg/mL).

#### 2.1.4. Effects of FSGLR (S7) and GIEWA (S10) on Expression Levels of Genes Involved in Lipid Metabolism in OA-Induced NAFLD Cell Model

**Figure 4:**
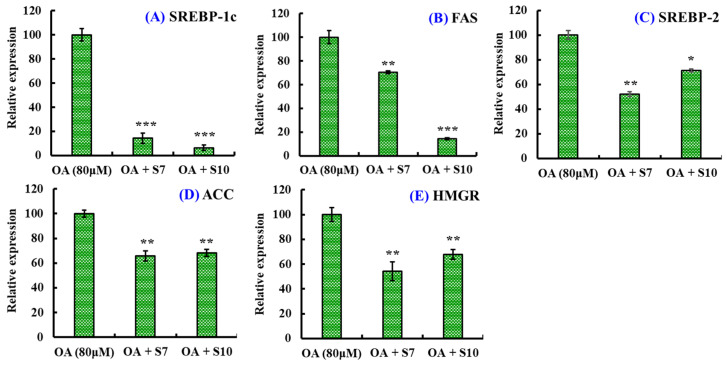
Effects of FSGLR (S7) and GIEWA (S10) on the mRNA expression levels of lipid synthesis in OA-induced HepG2 cells. (**A**) SREBP-1c; (**B**) FAS; (**C**) SREBP-2; (**D**) ACC; (**E**): HMGR. *** *p* < 0.001, ** *p* < 0.01, and * *p* < 0.05 vs. model group.

Figure 4 shows that FSGLR (S7) and GIEWA (S10) could significantly reduce the gene expression levels of sterol-regulatory element-binding protein-1c (SREBP-1c), fatty acid synthase (FAS), SREBP-2, acetyl-CoA carboxylase (ACC), and hydroxymethylglutaryl-CoA reductase (HMGR) compared with the OA-induced HepG2 cells (*p* < 0.05, 0.01, or 0.001). In addition, GIEWA (S10) showed a stronger ability to regulate the mRNA expression of SREBP-1c and FAS, but FSGLR (S7) showed a greater ability to regulate the mRNA expression of SREBP-2, ACC, and HMGR.

**Figure 5:**
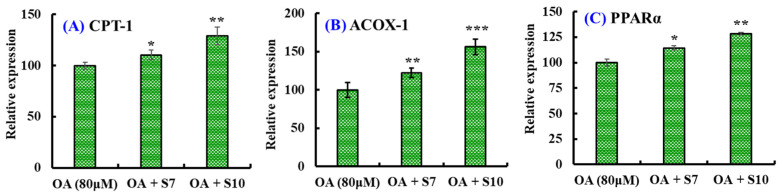
Effects of FSGLR (S7) and GIEWA (S10) on the mRNA expression levels of lipid β-oxidation in OA-induced NAFLD cell model. (**A**) CPT-1; (**B**) ACOX-1; (**C**) PPARα. *** *p* < 0.001, ** *p* < 0.01, and * *p* < 0.05 vs. model group.

In addition, FSGLR (S7) and GIEWA (S10) could significantly increase the mRNA expression levels of carnitine palmitoyltransferase 1 (CPT-1), acyl-CoA oxidase 1 (ACOX-1), and PPARα compared with the model cells without BPs (*p* < 0.05, 0.01 or 0.001). Moreover, GIEWA (S10) showed a stronger ability to regulate genes involved in the oxidative degradation of lipids than FSGLR (S7) (Figure 5).

### 2.2. Antioxidant Activity of FSGLR (S7) and GIEWA (S10) in OA-Induced NAFLD Cell Model

#### 2.2.1. Effects of FSGLR (S7) and GIEWA (S10) on ROS Levels in OA-Induced NAFLD Cell Model

**Figure 6:**
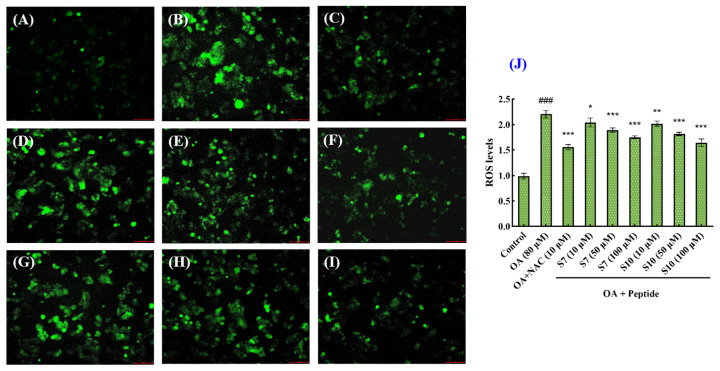
Effects of FSGLR (S7) and GIEWA (S10) on ROS levels of OA-induced NAFLD cell model at 10, 50, and 100 µM. (**A**) Control; (**B**) model (OA); (**C**) NAC; (**D**) S7 (10 μM); (**E**) S7 (50 μM); (**F**) S7 (100 μM); (**G**) S10 (10 μM); (**H**) S10 (50 μM); (**I**) S10 (100 μM); (**J**) ROS levels. ^###^ *p* < 0.001 vs. control group; *** *p* < 0.001, ** *p* < 0.01, and * *p* < 0.05 vs. model group.

Figure 6 shows that the ROS fluorescence intensity of the OA-induced HepG2 cells was significantly higher than that of the control group, whereas the ROS fluorescence intensity of the groups pretreated with NAC, FSGLR (S7), or GIEWA (S10) showed varying degrees of decrease compared to the model group, indicating that the ROS levels were significantly reduced by FSGLR (S7) and GIEWA (S10) pretreatment in comparison to the model group. Furthermore, the quantitative analysis showed that, when the FSGLR (S7) and GIEWA (S10) concentrations were in the range of 10 μM to 100 μM, the ROS levels in HepG2 cells induced by OA were gradually decreased in a dose-dependent manner (*p* < 0.05, 0.01, or 0.001) (Figure 6J). At 100 μM, the ROS content in the FSGLR (S7) and GIEWA (S10) groups decreased from 2.20 ± 0.060 to 1.75 ± 0.022 and 1.65 ± 0.060, respectively. In addition, GIEWA (S10) showed a stronger ability to decrease the ROS levels than FSGLR (S7) at the studied concentrations, which indicated that FSGLR (S7) and GIEWA (S10) could protect the HepG2 cells against OA-induced oxidative damage.

#### 2.2.2. Effects of FSGLR (S7) and GIEWA (S10) on Antioxidant Activity in OA-Induced NAFLD Cell Model

**Figure 7:**
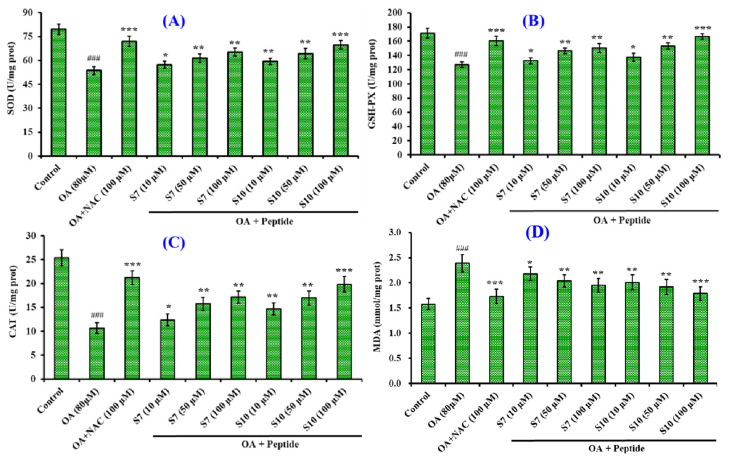
Effects of FSGLR (S7) and GIEWA (S10) on SOD (**A**), GSH-Px (**B**), CAT (**C**), and MDA (**D**) activity in OA-induced NAFLD cell model. ^###^ *p* < 0.05 vs. control group; *** *p* < 0.001, ** *p* < 0.01, and * *p* < 0.05 vs. model group. The cells were collected and lysed, and then the SOD, GSH-Px, CAT, and MDA content was determined.

Figure 7 shows that the activity of superoxide dismutase (SOD), glutathione peroxidase (GSH-Px), and catalase (CAT) induced by OA in HepG2 cells was gradually increased, while MDA production was gradually decreased as the FSGLR (S7) and GIEWA (S10) concentrations increased from 10 μM to 100 μM. At 100 μM, the SOD activity in the FSGLR (S7) and GIEWA (S10) groups was increased remarkably from 53.64 ± 2.47 U/mg prot to 65.28 ± 2.57 and 69.78 ± 2.66 U/mg prot, respectively (*p* < 0.01); the GSH-Px activity in the FSGLR (S7) and GIEWA (S10) groups was increased remarkably from 126.83 ± 3.96 U/mg prot to 150.29 ± 6.24 and 166.74 ± 3.82 U/mg prot, respectively (*p* < 0.01); the CAT activity in the FSGLR (S7) and GIEWA (S10) groups was increased remarkably from 10.66 ± 1.12 U/mg prot to 17.16 ± 1.29 and 19.86 ± 1.68 U/mg prot, respectively (*p* < 0.01); and the production of MDA in the FSGLR (S7) and GIEWA (S10) groups was decreased to 1.95 ± 0.134 and 1.79 ± 0.136 nmol/mg prot, respectively. Moreover, the content in the FSGLR (S7) and GIEWA (S10) groups was notably lower than the level of MDA production in the model group (2.39 ± 0.173 nmol/mg prot) (*p* < 0.01). Furthermore, GIEWA (S10) showed a stronger impact in terms of increasing the antioxidant activity than FSGLR (S7) at all studied concentrations.

### 2.3. Molecular Docking Analysis

**Figure 8:**
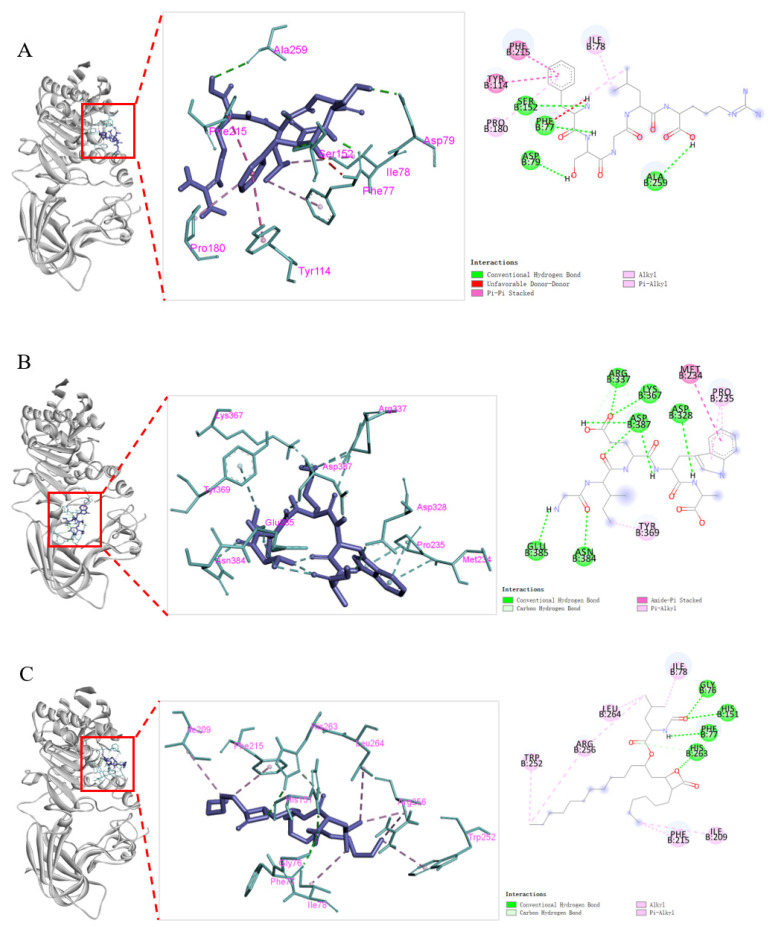

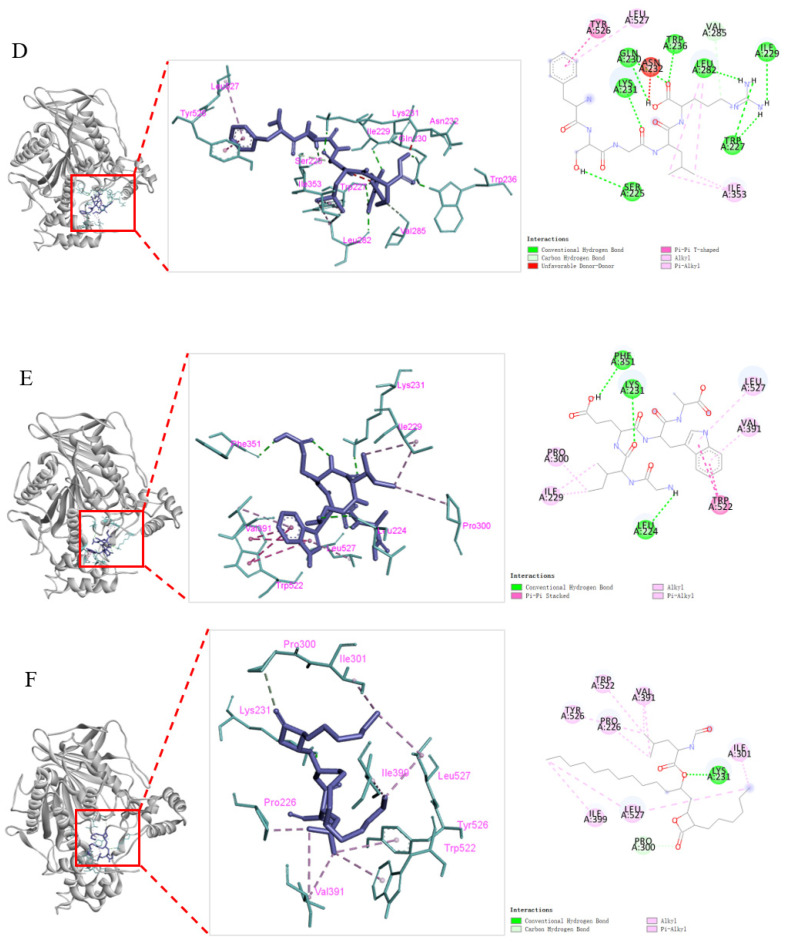
Molecular docking of FSGLR (S7), GIEWA (S10), and Orlistat with pancreatic lipase and cholesterol esterase. (**A**): S7-1LPB; (**B**): S10-1LPB; (**C**): Orlistat-1LPB; (**D**): S7-1F6W; (**E**): S10-1F6W; (**F**): Orlistat-1F6W.

To further explore the binding relationship between pancreatic lipase (PL) or cholesterol esterase (CE) and FSGLR (S7) or GIEWA (S10), molecular docking was performed (Figure 8). The binding affinity of FSGLR (S7) and GIEWA (S10) with pancreatic lipase (PL) was −7.3 and −7.1 kcal/mol, which was similar to that of Orlistat (−7.0 kcal/mol). In addition, the binding affinity of FSGLR (S7) and GIEWA (S10) with cholesterol esterase (CE) was −8.4 and −8.5 kcal/mol, showing a stronger affinity for lipase than Orlistat (−6.4 kcal/mol) (Table 1).

In the structure of PL (PDB ID: 1LPB), the catalytic triad of Ser152-His263-Asp176 and the hydrophobic pocket consisting of Phe77, Ile209, Pro180, Tyr114, and Phe215 play key roles in inhibiting pancreatic lipase activity [45]. Figure 8C shows that Orlistat formed hydrogen bonds with the amino acid residues Gly76 and His263 in 1LPB and the active sites His151 and Phe77 in the surface loop. It formed hydrophobic interactions with Trp252, Arg256, Leu264, Ile78, Phe215, and Ile209. FSGLR (S7) formed hydrogen bonds with amino acid residue Asp79 and active sites Ser152, Phe77, and Ala259 in the surface loop of 1LPB. Hydrophobic interactions were formed with Pro180, Tyr114, Phe215, and Ile78. Among them, Phe77, Phe215, and Ile78 were the amino acid residues shared by FSGLR (S7) and Orlistat when they bound to pancreatic lipase (Figure 8A). GIEWA (S10) formed hydrogen bonds with amino acid residues Asn384, Glu385, Asp328, Asp387, Lys367, and Arg337 in 1LPB. It formed a hydrophobic interaction with Pro235, Tyr39, and Met234 (Figure 8B). GIEWA (S10) interacted with 1LPB mainly through hydrogen bonding, where Asp328 and Asp387 were also the main hydrogen bonding sites between protein hydrolyses and 1LPB obtained by Zhang et al. [46].

Hydrogen bonds and hydrophobic forces are important in maintaining the stability of protein molecules; the formation of hydrogen bonds enhances the intermolecular interaction; and hydrophobic forces play a major role in maintaining protein conformation. Figure 8F shows that Orlistat formed hydrogen bonds with amino acid residue Lys231 and hydrophobic interactions with Tyr526, Trp522, Pro226, Ile399, Val391, Leu527, and Ile301 of CE (PDB ID: 1F6W). FSGLR (S7) formed hydrogen bonds with amino acid residues Gln230, Lys231, Ser225, Trp236, Leu282, Trp227, and Ile229 of 1F6W. Hydrophobic interactions were formed with Tyr526, Leu527, and Ile353 (Figure 8D). Moreover, Lys231, Tyr526, and Leu527 were the amino acid residues shared by FSGLR (S7) and Orlistat when they bound to cholesterol esterase. GIEWA (S10) formed hydrogen bonds with amino acid residues Lys231, Phe351, and Leu224 in 1LPB. It formed hydrophobic interactions with Pro300, Ile229, Leu527, Val391, and Trp522 (Figure 8E). It is noteworthy that Lys231, Leu527, Val391, and Trp522 were the common amino acid residues shared by GIEWA (S10) and Orlistat in binding to cholesterol esterase.

The Keap1-Nrf2 pathway is an important pathway for cellular oxidative stress. Keap1, as an inhibitor of Nrf2, renders Nrf2 inactive when the two are combined, and the Kelch region, as one of the five regions of the Keap1 protein, plays a key role in coupling with Nrf2. The Kelch domain is divided into five subpockets, P1 (Arg415, Arg483, Gly462, Ile461, Phe478, and Ser508), P2 (Arg380, Asn382, Asn414, and Ser363), P3 (Ala556, Gly509, Gly571, Gly603, Ser555, and Ser602), P4 (Gln530, Ser555, and Tyr525), and P5 (Phe577, Ser602, Tyr334, and Try572) [47].

**Figure 9:**
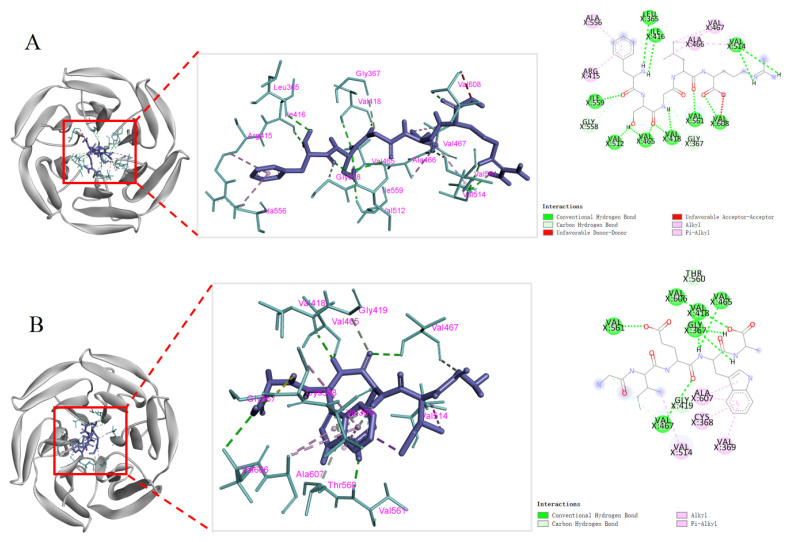
Molecular docking of FSGLR (S7) and GIEWA (S10) with Kelch region of Keap1 protein. (**A**): S7-2FLU; (**B**): S10-2FLU.

The molecular docking of FSGLR (S7) and GIEWA (S10) with the Kelch region (PDB ID: 2FLU) of the Keap1 protein is shown in Figure 9. The binding energies of FSGLR (S7) and GIEWA (S10) with the Kelch region were −9.5 kcal/mol and −8.6 kcal/mol, respectively (Table 2). Figure 9A shows that FSGLR (S7) formed hydrogen bonds with amino acid residues Ile559, Ile416, Leu365, Val512, Val465, Val418, Val561, Val608, and Val514 of 2FLU. It formed hydrophobic interactions with Arg415 in subpocket P1 and Ala556 in subpocket P3, as well as Val467 and Ala466. GIEWA (S10) formed hydrogen bonds with amino acid residues Gly367, Val561, Val606, Val418, Val465, and Val467 in 2FLU. Hydrophobic interactions were formed with Cys368, Ala607, and Val369 (Figure 9B). Among them, Gly367, Val561, Val606, Val418, Val465, and Val514 were also the binding sites of potential Keap1 inhibitors to 2FLU studied by Vellur et al. [48].

## 3. Discussion

### 3.1. NAFLD and OA-Induced NAFLD Cell Model

NAFLD is the most common cause of chronic liver disease worldwide and encompasses a wide disease spectrum, including NASH, fibrosis, and cirrhosis [49]. Presently, there are several hypotheses that account for NAFLD’s pathogenesis, and the “two-hit” hypothesis is one of the dominant theories used to discuss NAFLD [50]. In brief, lipid deposition in the liver causes hyperglycosemia and hyperlipemia, and the hepatic cells initially develop steatosis, which is called the “first hit”. The “second hit” is thought to be oxidative damage caused by lipid accumulation and the subsequent inflammatory responses and fibrosis. Thus, reducing the lipid deposition and oxidative stress levels in the liver is essential in ameliorating NAFLD [4,8].

Lipid accumulation in liver cells induces oxidative stress, inflammation, more fibrogenic cytokines, and apoptosis [51,52]. It also highlights the key role of FFAs in the pathogenesis of NAFLD and NASH [53,54]. HepG2 is a human hepatoma cell line and displays many differentiated hepatic functions, such as TC and TG metabolism, glycogen and bile acid synthesis, the secretion of plasma proteins, lipoprotein metabolism and transport, and insulin signaling [55]. Therefore, HepG2 cells are believed to be a suitable cell model to study the hypolipidemic and antioxidant functions related to the liver [56,57].

**Figure 10:**
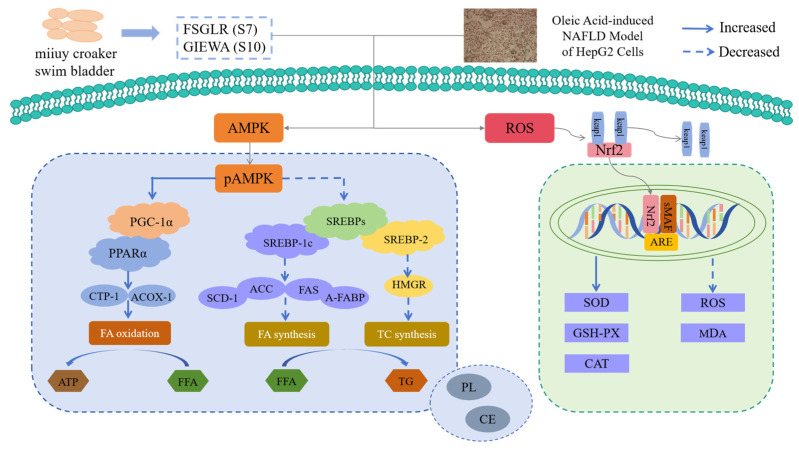
Functions of FSGLR (S7) and GIEWA (S10) in ameliorating OA-induced nonalcoholic fatty liver disease in HepG2 cells.

Therefore, we established the NAFLD cell model using OA-induced HepG2 cells based on the “two-hit” hypothesis, and this model was further used to explore the functions of FSGLR (S7) and GIEWA (S10) from the two perspectives of lipid lowering and antioxidant abilities (Figure 10). In addition, the MTT (cell viability), Oil Red O staining (lipid accumulation), and TG and TC content determination assays prove that the OA-induced NAFLD model of HepG2 cells is suitable for the study of the bioactivity and mechanisms of FSGLR (S7) and GIEWA (S10) (Figure 1 and Figure 2).

### 3.2. Functions of FSGLR (S7) and GIEWA (S10) in Ameliorating Lipid Metabolism

Modern unhealthy lifestyles, especially a HFD and lack of exercise, lead to the large accumulation of lipids in the human body, and the excess FFAs overloaded in the body are transferred from the bloodstream to the liver. If the rate of lipid oxidation is far slower than the synthesis rate, the large amounts of lipids accumulated in the liver will result in lipid metabolism disorder, steatosis, and ultimately the development of NAFLD [4,58]. In this context, VFVRN derived from chickpea protein hydrolysate showed strong hypolipidemic activity and could regulate lipid metabolism via regulating the LXRα/SREBPs/HMGR pathway [59,60]. Antioxidant peptides of VIAPW and IRWWW from *Miichthys miiuy* muscle could decrease the content of TG and TC via regulating the AMP-activated protein kinase (AMPK) pathway [58,61]. In our experiment, FSGLR (S7) and GIEWA (S10) could remarkably lower the lipid quantity in OA-induced HepG2 cells to reduce the probability of NAFLD occurrence.

TG provides much of the energy needed for human tissues to function, and TC is critical in building and maintaining key parts of cells. However, blood containing high levels of TG and TC will significantly raise the likelihood of a variety of cardiovascular and cerebrovascular diseases [62]. Therefore, the ability to reduce the TG and TC content becomes a key index in the evaluation of hypolipidemic components. Figure 2 indicates that FSGLR (S7) and GIEWA (S10) could dose-dependently decrease the TG and TC content of OA-induced HepG2 cells. Bile salt binding and PL- and CE-inhibitory abilities are important indicators of in vitro hypolipidemic activity. Studies have shown that peptides can bind bile salts to intervene in the hepato-intestinal circulation and promote cholesterol conversion [63]. Pancreatic lipase can reduce the decomposition and absorption of dietary fat, and cholesterol esterase plays a role in lowering blood lipids by hydrolyzing cholesterol. Figure 3 shows that the IC_50_ values of FSGLR (S7) and GIEWA (S10) increased in a dose-dependent manner as the values of these three indices increased.

The molecular docking results showed (Figure 8 and Figure 9, Table 1 and Table 2) that the binding energies of FSGLR (S7) and GIEWA (S10) with the corresponding receptor proteins were at a low level. The two peptides mainly interacted with the receptor proteins through hydrogen bonding and hydrophobic interactions. Therefore, FSGLR (S7) and GIEWA (S10) may occupy some active binding sites and interact with each other to reduce the catalytic activity of PL and CE, on the one hand, and inhibit the binding of Keap1 and Nrf2, on the other hand, thereby achieving hypolipidemic and antioxidant effects.

In the liver, SREBP-1c and SREBP-2 regulate HMGR and ACC, which are the first enzymes for AMPK and key enzymes in the synthesis of fatty acids and TC [58,64]. ACC is an important rate-controlling enzyme that is responsible for the biosynthesis of malonyl-CoA, and malonyl-CoA is an essential precursor for fatty acid synthesis and can enhance the activity of FAS [58,64]. Moreover, malonyl-CoA can regulate CPT-1 to suppress lipid oxidation [65]. SREBP-2 can regulate HMGR to affect TC synthesis, and HMGR is the rate-limiting step in TC biosynthesis due to catalyzing the conversion of HMG-CoA into mevalonate [66]. Moreover, AMPK phosphorylates and inactivates the ACC enzyme, which can increase CPT-1 activity and stimulate lipid β-oxidation. The present study indicated that FSGLR (S7) and GIEWA (S10) could reduce the lipid quantity in OA-induced HepG2 cells by significantly downregulating the mRNA expression involved in lipid synthesis (Figure 4 and Figure 5).

PPARα is mainly distributed in tissues with high fat metabolism, such as the liver, heart, and kidneys. PPARα can affect the development of NAFLD and NASH because it is involved in the liver’s fatty acid oxidation [26,27]. CPT-1 is a downstream protein of PPARα and acts as key rate-limiting enzyme of fatty acid β-oxidation in the liver, and it can promote β-oxidation to reduce the intracellular FFAs and inhibit the secretion of cellular pro-inflammatory factors [26,27]. ACOX-1, which is enriched in the liver, increases with HFD and catalyzes the first step of β-oxidation [67]. Figure 5 indicates that FSGLR (S7) and GIEWA (S10) could upregulate the mRNA expression of CPT-1, ACOX-1, and PPARα to accelerate the β-oxidation of FFAs, which further reduced the lipid, TC, and TG levels.

Therefore, the hypolipidemic functions of FSGLR (S7) and GIEWA (S10) are related to the reduction in the mRNA expression of hypolipidemic factors and improvements in the mRNA expression of lipolysis factors (Figure 10).

### 3.3. Functions of FSGLR (S7) and GIEWA (S10) in Regulating Intracellular Antioxidant System

Superfluous ROS in the liver results in cellular dysfunction and is regarded as a vital factor leading to NAFLD [68]. In our experiment, the ROS levels in the OA-induced HepG2 cells observably exceeded those in the control group (*p* < 0.001) (Figure 6). Under normal homeostasis, cells can clear physiological ROS in a timely manner via antioxidative enzymes (such as SOD, CAT, and GSH-Px) and antioxidants (such as vitamins A/C/E and GSH) [69,70]. In NAFLD, overproduced ROS oxidize FFAs, and these oxidized FFAs enter the liver cells to destroy the mitochondrial electron transport chain and intracellular antioxidant system, which further leads to a decrease in the activity of intracellular antioxidases, promotes the mass production of ROS, and increases the production of lipid peroxide (MDA) [8,68]. In addition, oxidative stress can also activate inflammatory pathways and cause mitochondrial dysfunction. These destructive effects lead to the deterioration of NAFLD into NASH [8].

Peptides (MW < 1 kDa) from monkfish could increase the antioxidative activity in the liver to prevent NAFLD progression by means of regulating the AMPK/Nrf2 pathway and intestinal flora [71,72]. High-Fischer-ratio oligopeptides from hard-shelled mussels showed significant hepatoprotective activity by initiating the intracellular antioxidant system and suppressing liver inflammation in mice [73]. In our experiment, we determined the activity of antioxidases and the content of lipid peroxides, and the findings showed that FSGLR (S7) and GIEWA (S10) could dose-dependently reduce the ROS level (Figure 6), significantly increase the activity of SOD, GSH-PX, and CAT, and reduce the MDA content (Figure 7) in the OA-induced NAFLD cell model. It was proven that FSGLR (S7) and GIEWA (S10) exerted strong cytoprotective effects on the HepG2 cells against OA-induced oxidative damage (Figure 10). These results indicate that regulating the antioxidant response could be a promising method to prevent and cure NAFLD [8,68].

The pathogenesis of NAFLD is very complex, involving lipid accumulation, oxidative stress, inflammation, apoptosis, and many other aspects. However, it is difficult to explain the mechanism of action of a compound by simply detecting the hypolipidemic and antioxidant activity in a cellular experiment. In our subsequent animal experiments, we will expand the detection range so that we can better explain the roles of FSGLR (S7) and GIEWA (S10) in ameliorating NAFLD at the animal level.

## 4. Materials and Methods

### 4.1. Materials and Chemical Reagents

Dulbecco’s modified eagle medium (DMEM), fetal bovine serum (FCS), bovine serum albumin (BSA), L-glutamine, OA, SV, β-actin, and MTT were purchased from Sigma-Aldrich (Shanghai) Trading Co., Ltd. (Shanghai, China). SOD, GSH-Px, CAT, MDA, TC, TG, Oil Red O staining, and bicinchoninic acid (BCA) protein assay kits were purchased from the Nanjing Jiancheng Bioengineering Institute (Nanjing, Jiangsu, China). Sodium taurocholate, pancreatic lipase, cholesterol esterase, and Tris buffer solution were purchased from Solarbio Life Sciences Co., Ltd. (Beijing, China). Orlistat was purchased from Targetmol Chemicals Co., Ltd. (Boston, MA, USA). P-nitrophenyl butyrate (PNPB) was purchased from Shanghai Macleans Biochemical Co., Ltd. (Shanghai, China). FSGLR (S7) and GIEWA (S10) were synthesized by Shanghai Apeptide Co., Ltd. (Shanghai, China). The purity of the synthesized peptides was higher than 98%, as determined via the HPLC method. The molecular mass of the synthesized peptides was confirmed by the manufacturer using electrospray ionization mass spectrometry (ESI-MS).

### 4.2. HepG2 Cell Culture and Establishment of OA-Induced NAFLD Model of HepG2 Cells

HepG2 human hepatoma cell line was purchased from Shanghai Cell Bank, Chinese Academy of Sciences. HepG2 cells were cultured in DMEM containing 50 mg/mL streptomycin, 50 U/mL penicillin, 2 mM glutamine, and 10% FCS in a humidified atmosphere of 5% CO_2_ at 37 °C.

The OA-induced NAFLD cell model was established according to previous methods [74,75]. HepG2 cells in the logarithmic growth phase were seeded into 96-well plates and cultivated in a humidified atmosphere of 5% CO_2_ at 37 °C for 24 h. OA was dissolved in 10% BSA and added to the DMEM with a final concentration of 80 μM. After this, the cells were cultured for 24 h, and the morphologies of the HepG2 cells were observed and photographed using a microscope. The HepG2 cells in the control wells were cultured for 24 h in DMEM without OA.

### 4.3. Determination of Cell Viability, TC, TG, MDA, and Antioxidant Enzymes

The viability of the HepG2 cells was evaluated using the MTT assay via a previous method [71]. In brief, S7 and S10 were dissolved in growth medium to form a peptide solution with a concentration of 200 μM. Then, the peptide solution was added to the growth medium of HepG2 cells with final concentrations of 10, 50, and 100 μM, and the cells were incubated for 24 h with peptide administration. The control group was treated with the growth medium without peptides. SV (1.0 mM) was used as a positive control.

The intracellular TC, TG, and MDA content and activity of SOD, GSH-Px, and CAT were measured using the relevant assay kits, according to the manufacturer’s instructions [76,77].

### 4.4. Oil Red O Staining Assay

This assay was performed according to the Oil Red O staining kit, following the manufacture’s instructions [28]. HepG2 cells were fixed on 96-well plates with 4% formaldehyde for 30 min, washed with phosphate-buffered saline (PBS) (pH 7.0) twice, and rinsed with 60% isopropanol for 10 min. After this, the HepG2 cells were incubated in 3% Oil Red O solution for 1 h and rinsed with PBS thrice to remove the unbound dye. After staining, the cells were dissolved in DMSO, and they were transferred to a new 96-well plate. Then, the absorbance at 358 nm of the stained cells was measured using a microplate reader. Images of stained cells were taken using an Olympus IX71 inverted microscope (Olympus Co., Ltd., Shinjuku, Japan).

### 4.5. In Vitro Hypolipidemic Activity Analysis

The in vitro bile salt binding rate was determined using the method of Zhang et al. [46], with slight modifications. First, 1 mL of hydrochloric acid (0.01 M) was added to 1 mL of polypeptide (0.5 mg/mL) and incubated at 37 °C for 1 h. After adjusting the pH to 6.4, 5 mL bile salt solution (1 mM) was added. After 1 h of reaction at 37 °C, 2.5 mL of the supernatant was centrifuged and collected, 7.5 mL 60% sulfuric acid was added, and the mixture was reacted at 70 °C for 30 min. Finally, the absorbance was measured at 387 nm. The blank group was treated with 0.1 M phosphate buffer instead of bile salts. The bile acid binding rate was calculated using the following formula:

Bile salt binding rate (%) = [(Ab − As)/Ab] × 100%(1)

where As and Ab are the absorbance values determined at 387 nm of the sample and the blank after the reaction, respectively.

The in vitro pancreatic lipase (PL) inhibitory activity was measured using the method of Cai et al. [78], with slight modifications. Pancreatic lipase was first dissolved in 100 mM Tris buffer (2 mg/mL) and the supernatant was obtained by centrifugation. Tris buffer was then prepared as 0.1% (*w*/*v*) PNPB solution. The 50 μL peptide sample was incubated with 50 μL Tris buffer solution and 50 μL pancreatic lipase solution at 37 °C for 15 min, and then 100 μL PNPB solution was added. After 20 min at 37 °C, the absorbance value was measured at 405 nm. In the blank group, pure water was used instead of a sample. In the blank control group, the samples were replaced with pure water and the pancreatic lipase solution was replaced with a buffer solution. The sample control group was treated with Tris buffer instead of the pancreatic lipase solution. The pancreatic lipase (PL) inhibitory rate was calculated using the following formula:

PL inhibitory rate (%) = [1 − (A − A0)/(B − B0)] × 100%(2)

where A, A0, B, and B0 are the absorbance values determined at 405 nm of the sample, the sample control, the blank, and the blank control after the reaction, respectively.

The in vitro cholesterol esterase (CE) inhibitory activity was measured using the method of Jafar et al. [79], with slight modifications. Buffers were first prepared with sodium chloride (0.1 M), p-nitrophenyl butyrate (PNPB 0.2 mM), sodium taurocholate (5.16 mM), and sodium phosphate buffer (0.1 M). Then, 10 μL PNPB solution, 25 μL peptide sample, and 50 μL cholesterol esterase solution were added to 1 mL buffer, and, after reaction at 25 °C for 5 min, the supernatant was centrifuged and the absorbance was measured at 405 nm. Cholesterol esterase was replaced with pure water in the background control group, with pure water in the sample control group, and with pure water in the blank control group. The cholesterol esterase (CE) inhibitory rate was calculated using the following formula:

CE inhibitory rate (%) = [1 − (A − B)/(C − D)] × 100%(3)

where A, B, C, and D are the absorbance values determined at 405 nm of the sample, the background control, the sample control, and the blank control after the reaction, respectively.

### 4.6. Intracellular ROS Level Analysis

The ROS level was determined by the dichlorodihydrofluorescein diacetate (DCFH-DA) staining method [77]. Briefly, HepG2 cells were seeded in 12-well plates and DCFH-DA was diluted to a final concentration of 10 μM at a ratio of 1:1000. Next, after the removal of the cell culture medium, the cells were washed three times with PBS. After this, the HepG2 cells were incubated with 10 μM DCFH-DA for 30 min at 37 °C. After washing with PBS three times again, the fluorescence of the HepG2 cells was monitored at 485 nm excitation (535 nm emission). Finally, images were acquired using a fluorescence microscope to observe the fluorescence intensity of DCFH-DA.

### 4.7. Molecular Docking

The PL protein (PDB ID: 1LPB), CE protein (PDB ID: 1F6W), and Keap1 protein (PDB ID: 2FLU) were downloaded from the Protein Database (https://www.rcsb.org) (accessed on 23 July 2024), and the proteins were dehydrated and hydrogenated. The ChemDraw 3D software (version 20.0) was used to draw the 3D structure of the small molecules and add hydrogen atoms to it. The AutoDock software (version 1.5.6) was used to perform molecular docking between the proteins and small molecules, and the docking results were viewed. The results were visualized and analyzed with the Discovery Studio software (version 19.1).

### 4.8. Preparation of Protein Extract of HepG2 Cells

After washing twice with PBS, a lysis buffer (1% Triton X-100, 1% deoxycholate, 0.1% SDS) was added to HepG2 cells on ice for 20 min. Then, the mixtures were centrifuged at 12,000× *g* at 4 °C for 20 min. The protein concentration was determined using a BCA protein assay kit [80].

### 4.9. Fluorescence Quantitative PCR Analysis

The RNA extraction process was performed according to previous methods [28,56]. RNA extraction: HepG2 cells (2.5 × 10^5^ cells/well) in 12-well plates (1 mL) were incubated in DMEM (containing 10% FCS) for two days. The HepG2 cells were washed twice using PBS and incubated in 500 μL of FCS-free DMEM (containing 1.0% (*w*/*v*) BSA) for two days, with or without 0.75 mM sodium oleate or sodium oleate plus samples (100 μg/mL). After culture, the HepG2 cell culture plates were placed on ice, the cell culture media were wiped off, and they were rinsed with ice-cold Tris-buffered saline (TBS). Treated HepG2 cells from each disc were lysed in TBS (150 μL, containing 0.02% Triton-X100 detergent) and were moved to 1.5 mL tubes. To facilitate cell lysis, the tubes with HepG2 cells were frozen using liquid nitrogen and thawed quickly in a 37 °C water bath; this process of cell lysis was repeated a couple of times. The total RNA was separated from the cell lysates using TRIzol (Thermo Fisher Scientific (China) Co., Ltd., Shanghai, China). The total amount and purity of RNA were measured using a NanoDrop 2000/2000c spectrophotometer (Thermo Fisher Scientific (China) Co., Ltd., Shanghai, China). The RNA was reverse-transcribed into cDNA using a TransStart Top Green qPCR SuperMix kit with fluorescence labeling with SYBR Green, according to the manufacturer’s instructions (TransGen Biotech Co., Ltd., Beijing, China). Amplification was carried out using a three-step temperature cycle in a 10 μL reaction system as follows: pre-degenerated at 95 °C for 1 min, degenerated at 95 °C for 20 s, renaturated at 58 °C for 30 s, and extended at 72 °C for 10 s with 39 cycles. The specificity of the PCR was verified by a melting curve analysis from 72 °C to 95 °C. The GADPH gene was chosen as the internal reference, while the threshold and Ct (threshold cycle) values acquired via RT PCR were used to analyze the genes’ mRNA levels according to the 2^−ΔΔCt^ method. All data were normalized and are presented as the mean ± SD (*n* = 3). The data were processed in the SPSS software (version 27.0). The forward and reverse PCR primers are shown in Table 3.

### 4.10. Statistical Analysis

Data were expressed as means ± standard deviation (SD) (*n* = 3). An ANOVA test was used to compare the mean values of each treatment. Significant differences (*p* < 0.05, *p* < 0.01, or *p* < 0.001) were analyzed using Duncan’s multiple range test.

## 5. Conclusions

In summary, FSGLR (S7) and GIEWA (S10) from miiuy croaker swim bladders were proven to be functional molecules in inhibiting lipid accumulation in OA-induced HepG2 cells, and their hypolipidemic functions were related to their bile salt binding abilities, PL and CE activity, and adipokine mRNA expression. In addition, FSGLR (S7) and GIEWA (S10) could significantly protect HepG2 cells against OA-induced oxidative damage, and their antioxidant functions were related to the activity of intracellular antioxidant proteases, as well as the production of lipid peroxides in the cells. Moreover, the hypolipidemic and antioxidant effects of FSGLR (S7) and GIEWA (S10) were also validated by molecular docking. The present study was limited to the lipid-lowering and antioxidant activity of FSGLR (S7) and GIEWA (S10) at the cellular level in an NAFLD model. In subsequent experiments, we will focus on S7 and S10 in in vivo studies and further explore their practical application as therapeutic candidates for NAFLD.

## Figures and Tables

**Table 1:** The potential binding sites of FSGLR (S7), GIEWA (S10), and Orlistat with 1LPB and 1F6W.

Ligand	Binding Energy with 1LPB (kcal/mol)	Hydrogen Bonds	Hydrophobic Interactions	Binding Energy with 1F6W (kcal/mol)	Hydrogen Bonds	Hydrophobic Interactions
S7 (FSGLR)	−7.3	Ser152, Phe77, Asp79, Ala259	Phe215, Tyr114, Pro180, Ile78	−8.4	Gln230, Lys231, Ser225, Trp236, Leu282, Trp227, Ile229	Tyr526, Leu527, Ile353
S10 (GIEWA)	−7.1	Asn384, Lys367, Asp328, Asp387, Glu385, Arg337	Pro235, Tyr369, Met234	−8.5	Phe351, ys231, Leu224	Pro300, Ile229, Leu527, Val391, Trp522
Orlistat	−7.0	Gly76, His151, Phe77, His263	Trp252, Arg256, Leu264, Ile78, Phe215, Ile209	−6.4	Lys231	Tyr526, Trp522, Pro226, Ile399, Val391, Leu527, Ile301

**Table 2:** The interaction sites of FSGLR (S7) and GIEWA (S10) with 2FLU.

Ligand	Binding energy with 2FLU (kcal/mol)	Hydrogen Bonds	Hydrophobic Interactions
S7 (FSGLR)	−9.5	Ile559, Ile416, Leu365, Val512, Val465, Val418, Val561, Val608, Val514	Ala556, Ala466, Val467, Arg415
S10 (GIEWA)	−8.6	Gly367, Val561, Val606, Val418, Val465, Val467	Cys368, Ala607, Val514, Val369

**Table 3:** The primers used for gene amplification.

Primer	Sequence (5′−3′)
ACC-F	TGATGTCAATCTCCCCGCAGC
ACC-R	TTGCTTCTTCTCTGTTTTCTCCCC
SREBP-1c-F	CCATGGATGCACTTTCGAA
SREBP-1c-R	CCAGCATAGGGTGGGTCAA
SREBP-2-F	CTGCAACAACAGACGGTAATGA
SREBP-2-R	CCATTGGCCGTTTGTGTCAG
FAS-F	CGGTACGCGACGGCTGCCTG
FAS-R	GCTGCTCCACGAACTCAAACACCG
HMGR-F	GGACCCCTTTGCTTAGATGAAA
HMGR-R	CCACCAAGACCTATTGCTCTG
CPT1-F	CGTCTTTTGGGATCCACGATT
CPT1-R	TGTGCTGGATGGTGTCTGTCTC

## Data Availability

The supporting data for this study are available within the article.
